# Cessation support for smokers with mental health problems: a survey of resources and training needs

**DOI:** 10.1016/j.jsat.2017.06.008

**Published:** 2017-09

**Authors:** Erikas Simonavicius, Debbie Robson, Andy McEwen, Leonie S. Brose

**Affiliations:** a Department of Addictions, Institute of Psychiatry, Psychology and Neuroscience, King's College London, London, United Kingdom; b National Centre for Smoking Cessation and Training, 1 Great Western Industrial Centre, Dorchester, United Kingdom; c UK Centre for Tobacco and Alcohol Studies, United Kingdom

**Keywords:** Smoking cessation, Mental health, Mental disorders, Stop smoking services, Preventive health services

## Abstract

**Aims:**

Around thirty percent of smokers have a mental health problem. Smoking cessation has been associated with mental health benefits, but smoking prevalence remains high in populations with mental health problems. This study aimed to assess mental health related knowledge, practice, and training needs of practitioners supporting smoking cessation.

**Methods:**

UK stop smoking practitioners (*n* = 717) recruited via a database of a national provider of smoking cessation training in June 2016 sufficiently completed an online survey about available resources, knowledge, confidence, and training needs related to smoking cessation and mental health. Responses were described and compared between practitioners with a mental health lead and those without such a lead in their service using chi-square statistics and *t*-tests.

**Results:**

A considerable proportion agreed (37%) or were undecided (28.9%) that smoking helped people with mental health problems feel better and agreed (17.2%) or were undecided (30.2%) that cessation would exacerbate mental health symptoms. Only 11.6% said their service had designated funding for smokers with mental health problems and 26.5% were or had a staff member who was a dedicated lead practitioner for mental health work. Practitioners from services that had a dedicated mental health lead were more confident in supporting smokers with different mental health problems and using different pharmacotherapies (all *p* < 0.001) and were more likely to disagree that cessation was detrimental (*p* = 0.001). A majority of practitioners were interested in training, particularly about smoking cessation effects on psychiatric medication (84.3% of *n* = 632) and how to tailor stop smoking support to clients with mental health problems (82.4%).

**Conclusion:**

Practitioners who support smoking cessation have limited knowledge about mental health and smoking but are willing to learn and improve. However, they are hindered by a lack of resources.

## Highlights

• Smokers with mental health problems benefit hugely from smoking cessation.
• However, these smokers encounter multiple barriers when looking for cessation support.
• Stop smoking clinics lack resources to provide tailored support for these smokers.
• Cessation practitioners want to expand their limited knowledge on mental health.

## 1 Introduction

Smoking prevalence in England has declined from 27% in 2000 to 16% in 2016 (Office for National Statictics, 2017). The improvement, however, has excluded people with mental health problems, where smoking rates have not significantly changed since the early 1990s (Szatkowski & McNeill, 2015) and remain twice as high as in the general population (Royal College of Physicians & Royal College of Psychiatrists, 2013). Among mental health patients, smoking contributes to reduced life expectancy (Royal College of Physicians & Royal College of Psychiatrists, 2013) and interferes with the metabolism of pharmacotherapy: smokers require higher doses of some psychiatric medication, which potentially increases medication side effects (Aguilar, Gurpegui, Diaz, & De Leon, 2005). Nevertheless, smokers with mental health problems are motivated to quit (Lukowski et al., 2015, Morris et al., 2009, Siru et al., 2009, Szatkowski and McNeill, 2015), willing to do so with specialist support (Kerr, Woods, Knussen, Watson, & Hunter, 2013), and quitting may improve their mental health (Banham and Gilbody, 2010, Campion et al., 2008, Stepankova et al., 2016, Taylor et al., 2014).

The need to focus on smoking alongside mental health treatment has been highlighted in national guidelines (Fiore et al., 2008, Hall and Prochaska, 2009, NICE, 2013) and covered by training programmes (Hiscock and Bauld, 2013, NCSCT, 2014b), but these smokers still encounter multiple barriers towards cessation. People with mental health problems smoke more intensely and are more nicotine dependent (McClave, McKnight-Eily, Davis, & Dube, 2010) which, along with the burden of ill health, makes quitting particularly challenging (Twyman, Bonevski, Paul, & Bryant, 2014). In psychiatry, where smoking was long considered a norm (Ratschen, Britton, & McNeill, 2011), patient smoking is usually overlooked by staff (Ratschen, Britton, Doody, Leonardi-Bee, & McNeill, 2009) or even facilitated during organised smoking breaks despite smoke-free legislation in mental health settings (Department of Health, 2006, Robson et al., 2016). Smokers with mental health problems also encounter difficulties in approaching specialist smoking cessation services. Mental health trusts rarely refer their patients to smoking cessation services (McNally and Ratschen, 2010, Parker et al., 2012), and in primary care these smokers are less frequently advised to quit than smokers without mental health problems (Szatkowski & McNeill, 2013). These disadvantages could stem from common misconceptions among healthcare professionals that smoking cessation aggravates mental health or that smokers with mental health problems lack confidence and motivation to quit (Kerr et al., 2013, Sheals et al., 2016).

In 1999 England established comprehensive smoking cessation services to support quitting, including among ‘the most disadvantaged smokers who want to quit’ (Department of Health, 1998, p. 28). The services support several hundred thousand stop smoking attempts every year (West, May, West, Croghan, & McEwen, 2013) with evidence-based pharmacological and behavioural interventions (NICE, 2008). In the services an array of professionals support smoking cessation, including but not limited to health and social care workers. Stop smoking practitioners are classified into ‘specialist’ and ‘community’: the former support smoking cessation as their main work role, and the latter provide smoking cessation support as a part of their job alongside their main role (e.g. practice nurses and pharmacists).

Cessation services consider smokers with mental health problems a targeted priority group (ASH, 2016, NCSCT, 2014a). To accommodate their needs, some services have a specific team member designated as a lead for smokers with mental health problems. Although the role is not standardised across all services, the designated leads within a smoking cessation service dedicate part of their time to supporting smokers with mental health problems, liaising with local mental health services, and providing training. In a survey of 27 Stop Smoking Services in London, half of services had a nominated lead for mental health work, but these practitioners on average spent only a quarter of their work time on supporting smokers with mental health problems (McNally & Ratschen, 2010). Also, there is little evidence about how well prepared practitioners are to support smokers who have mental health problems and how cessation support is tailored for these smokers (Gilbody et al., 2015). Therefore, this study aimed to:

1) Assess available resources for stop smoking practitioners to identify and support smokers with mental health problems.
2) Assess and compare practitioners' practice, attitudes, and confidence between those who have and do not have a staff member leading on mental health work.
3) Identify practitioners' knowledge and training needs.

## 2 Methods

### 2.1 Sample

The target population was all those who supported smoking cessation attempts as part of their professional role. As there is no central record of this group, a national training provider database was used to contact potential participants. In June 2016, an email invitation to complete an online questionnaire survey was sent to 22,214 email addresses, some outdated, of practitioners who at some point registered for recommended training at a UK national smoking cessation training provider database. Of those sent out, 4724 invitation emails were opened. Everyone who was sent an invitation to complete the survey had opted in to receive communications from the national smoking cessation training provider. Invitations were also distributed using newsletters of UK charity organisations focusing on smoking and public health. In total, 1056 respondents started the survey, of which 717 (67.9% of surveys started) sufficiently complete questionnaires (respondents were exposed to all survey items and answered > 80%) were used in the analyses. Completion of individual items was not mandatory, therefore, sample sizes range from 632 to 695 respondents due to missing data. Respondents in any sample were from the main study sample of N = 717.

### 2.2 Study design

The survey took approximately 10 to 15 min to complete. The survey was online for four weeks, two email reminders were sent two weeks and three days prior to its closure. Before starting a questionnaire, all participants were introduced to the research aims and assured of the anonymity and confidentiality of their responses. Participants could also choose to participate in a prize draw to win one of ten High Street vouchers, each worth £50, by providing contact details at the end of a questionnaire. Participants were ensured that their contact information will be kept and treated separately from the rest of survey data. Winners were randomly drawn from the 1056 who started the survey and provided their contact details.

### 2.3 Measures

The questionnaire was developed by the study authors based on the available guidelines and training on smoking cessation for smokers with mental health problems (Hiscock and Bauld, 2013, NCSCT, 2014b, NICE, 2013). The questionnaire was revised by a group of practitioners supporting smokers with mental health problems and an additional pilot survey was conducted with 11 practitioners.

The questionnaire consisted of 23 questions (some with several sub-questions) assessing four main areas:

1) Practitioners' demographic and professional characteristics (gender, age, country of residence, professional role, employment);
2) Services' resources and current practice supporting smokers with mental health problems (availability of funding and treatment manual for work with smokers who have mental health problems, and availability of a designated member of staff who leads work with this group of smokers);
3) Current support for smokers with mental health problems. This included i) current practice (how often practitioners ask clients about mental health, record mental health medication they are taking, and contact General Practitioners or mental health services when supporting a smoker with mental health problems), ii) practitioners' attitudes towards smokers with mental health problems assessed in comparison with smokers who do not have mental health problems (interest in smoking cessation, chances to quit smoking, dependence on nicotine, willingness to use smoking cessation medication, need to cut down smoking before quitting, and whether 4-week quit outcomes are equally appropriate for both groups) and iii) confidence in supporting these clients having different diagnoses (depression or anxiety, bipolar disorder, eating disorder, schizophrenia, other substance use disorder) or providing them different support (NRT, bupropion, varenicline, electronic cigarettes);
4) Practitioners' knowledge (proportion of smokers with mental health problems, interaction between smoking and psychiatric medication, knowledge about cessation support medication) and training needs when supporting smokers with mental health problems.

Survey items required a single answer or asked to indicate the level of agreement with a statement using a 5-point Likert scale. A list of all survey questions and answers is outlined in the appendix Table A1.

### 2.4 Analysis

IBM SPSS Statistics 22 was used for analyses. Descriptive statistics and frequencies were used to summarise practitioners' responses. Practitioners' experience in providing smoking cessation, estimates of proportions of smokers with mental health problems in the UK and in their practices, and confidence to support these smokers were described using means, standard deviations, and 95% confidence intervals. To compare practices, attitudes, confidence, knowledge, and training needs between practitioners who have and do not have a staff member leading on mental health work, chi square and independent sample *t*-tests were used. For statistically significant larger than 2 × 2 contingency tables, cells with adjusted residuals greater than ± 2.58 were identified as contributing to differences between groups (Sharpe, 2015).

## 3 Results

### 3.1 Demographic and work characteristics

Respondents were most likely to be 45 to 54 years old (34.7%), female (82.7%), working as a specialist (43.2%) or community (40.3%) practitioner, employed by a National Health Service (NHS) organisation (26.8%), general practice (25.5%) or pharmacy (18.4%) (Table 1), and had been providing smoking cessation support for an average of 6.2 years (SD = 4.3, 95% CI: 5.9–6.6).

**Table 1:** Respondents' demographic and work characteristics, N = 695.

Characteristic	Total, % (n)	No mental health lead, *n* = 515 (74.1%)	Mental health lead present, *n* = 180 (25.9%)	Comparison statistics
Gender, %(*n*)				
Male	17.3 (120)	16.3 (84)	20.0 (36)	χ^2^(1) = 1.3, *p* = 0.26
Female	82.7 (575)	83.7 (431)	80.0 (144)	

Age, % (*n*)				
Below 34	14.1 (98)	13.0 (67)	17.2 (31)	χ^2^(3) = 2.9, *p* = 0.40
35–44	22.2 (154)	21.6 (111)	23.9 (43)	
45–54	34.7 (241)	35.7 (184)	31.7 (57)	
55 or older	29.1 (202)	29.7 (153)	27.2 (49)	

Country of residence, % (*n*)				
England	94.7 (658)	94.8 (488)	94.4 (170)	χ^2^(1) = 0.03, *p* = 0.87
Scotland, Wales, or Northern Ireland	5.3 (37)	5.2 (27)	5.6 (10)	

Role				
Specialist practitioner^a^	43.2 (300)	41.2 (212)	48.9 (88)	**χ**^**2**^**(2) = 8.7, *p* = 0.013**
Community practitioner^b^	40.3 (280)	**43.5 (224)**	**31.1 (56)**	
Service manager, commissioner, or other	16.5 (115)	15.3 (79)	20.0 (36)	

Employer, % (n)				
NHS organisation	26.8 (186)	**21.2 (109)**	**42.8 (77)**	**χ**^**2**^**(5) = 56.3, *p* < 0.001**
General practice	25.5 (177)	**29.3 (151)**	**14.4 (26)**	
Pharmacy	18.4 (128)	**21.7 (112)**	**8.9 (16)**	
Local authority	11.2 (78)	**9.3 (48)**	**16.7 (30)**	
Company that runs stop smoking services	5.9 (41)	5.2 (27)	7.8 (14)	
Other or unknown	12.2 (85)	13.2 (68)	9.4 (17)	

Note: Bolded cells associated with adjusted residuals greater than ± 2.58 (α = 0.01).

a For specialist practitioners smoking cessation is their main work role.

b For community practitioners smoking cessation is not the main but a part of their job.

### 3.2 Resources

Over a quarter of practitioners had (17.4%) or were (9.1%) a lead for supporting smokers with mental health problems, and 11.6% said their service had funding for mental health work. More than a half of practitioners (57.3%) reported their service had a system to record clients' mental health status, and 16.2% had a manual guiding support for smokers with mental health problems.

### 3.3 Smoking cessation support for smokers with mental health problems

#### 3.3.1 Provision of support

More than two thirds of practitioners (69.1%) reported very often or always asking clients about their mental health, 75.5% were very often or always recording the medication clients were taking, and 35.5% were very often or always contacting other healthcare specialists regarding a cessation attempt (Table 2). Practitioners who had or were a lead for supporting smokers with mental health problems were more likely to always ask clients about their mental health status (χ^2^(4) = 22.1, *p* < 0.001), less likely to never record clients' mental health medication (χ^2^(4) = 10.3, *p* = 0.036), and less likely to never contact GPs or other healthcare services regarding a smoking cessation attempt of a client with mental health problems (χ^2^(4) = 22.6, *p* < 0.001) (Table 2).

**Table 2:** Provision of stop smoking support for smokers with mental health problems, N = 633.

	Total	No mental health lead, *n* = 462 (73%)	Mental health lead present, *n* = 171 (27%)	Comparison statistics
Ask about mental health status, % (*n*)				
Never	7.0 (44)	**8.7 (40)**	**2.3 (4)**	**χ**^**2**^**(4) = 22.1, *p* < 0.001**
Rarely	6.0 (38)	**7.4 (34)**	**2.3 (4)**	
Sometimes	17.9 (113)	19.3 (89)	14.0 (24)	
Very often	16.7 (106)	17.1 (79)	15.8 (27)	
Always	52.4 (332)	**47.6 (220)**	**65.5 (112)**	

Record mental health medication, % (*n*)				
Never	10.1 (64)	**12.1 (56)**	**4.7 (8)**	**χ**^**2**^**(4) = 10.3, *p* = 0.036**
Rarely	6.3 (40)	6.5 (30)	5.8 (10)	
Sometimes	8.1 (51)	6.9 (32)	11.1 (19)	
Very often	11.4 (72)	11.7 (54)	10.5 (18)	
Always	64.1 (406)	62.8 (290)	67.8 (116)	

Contact GP or mental health services, % (*n*)				
Never	22.9 (145)	**26.8 (124)**	**12.3 (21)**	**χ**^**2**^**(4) = 22.6, *p* < 0.001**
Rarely	15.3 (97)	14.1 (65)	18.7 (32)	
Sometimes	26.2 (166)	27.5 (127)	22.8 (39)	
Very often	10.9 (69)	9.1 (42)	15.8 (27)	
Always	24.6 (156)	22.5 (104)	30.4 (52)	

Note: Bolded cells associated with adjusted residuals greater than ± 2.58 (α = 0.01).

#### 3.3.2 Attitude towards smoking and mental health

When asked to compare smokers with and without mental health problems, the majority of respondents agreed (includes ‘strongly agree’ or ‘agree’ for the remainder of this paragraph) with evidence-based statements: 80.4% that smokers with mental health problems are more dependent on nicotine and 56.7% that they are less successful in cessation. In contrast to published evidence, 39.8% agreed that smokers with mental health problems are generally less interested in stopping smoking. In other comparisons, 53.2% agreed that smokers with mental health problems more often need to reduce smoking before quitting, 36.5% agreed that the standard 4-week quit outcome measure is equally appropriate for smokers with mental health problems, and 18.1% agreed that smokers with mental health problems are less willing to use cessation medication. Compared with those who worked in a service that had a mental health lead, practitioners in services without a lead were more likely to agree that smokers with mental health problems are less interested and less successful in quitting smoking than smokers without mental health problems (Table 3).

**Table 3:** Knowledge and attitude towards smokers with mental health problems compared with smokers without mental health problems; N = 688.

	Total	No mental health lead, *n* = 508 (73.8%)	Mental health lead present, *n* = 180 (26.2%)	Comparison statistics
Smoking helps smokers with mental health problems to feel better, % (*n*)				
Strongly disagree	12.1 (83)	11.4 (58)	13.9 (25)	χ^2^(4) = 5.3, *p* = 0.26
Disagree	22.1 (152)	20.7 (105)	26.1 (47)	
Neither agree nor disagree	28.9 (199)	29.9 (152)	26.1 (47)	
Agree	29.4 (202)	30.9 (157)	25.0 (45)	
Strongly agree	7.6 (52)	7.1 (36)	8.9 (16)	
Stopping smoking worsens symptoms of a mental health problem, % (*n*)				
Strongly disagree	15.3 (105)	**13.0 (66)**	**21.7 (39)**	**χ**^**2**^**(4) = 17.9, *p* = 0.001**
Disagree	37.4 (257)	35.2 (179)	43.3 (78)	
Neither agree nor disagree	30.2 (208)	32.5 (165)	23.9 (43)	
Agree	14.1 (97)	**16.1 (82)**	**8.3 (15)**	
Strongly agree	3.1 (21)	3.1 (16)	2.8 (5)	

Compared with smokers without mental health problems				
Smokers with mental health problems are less interested in stopping smoking, % (*n*)				
Strongly disagree	9.6 (66)	8.1 (41)	13.9 (25)	**χ**^**2**^**(4) = 14.2, *p* = 0.007**
Disagree	35.5 (244)	33.3 (169)	41.7 (75)	
Neither agree nor disagree	15.1 (104)	15.2 (77)	15.0 (27)	
Agree	32.0 (220)	**35.2 (179)**	**22.8 (41)**	
Strongly agree	7.8 (54)	8.3 (42)	6.7 (12)	
Smokers with mental health problems are less successful in quitting smoking, % (*n*)				
Strongly disagree	3.5 (24)	3.7 (19)	2.8 (5)	**χ**^**2**^**(4) = 20.5, *p* < 0.001**
Disagree	21.8 (150)	**18.1 (92)**	**32.2 (58)**	
Neither agree nor disagree	18.0 (124)	17.5 (89)	19.4 (35)	
Agree	49.4 (340)	52.0 (264)	42.2 (76)	
Strongly agree	7.3 (50)	8.7 (44)	3.3 (6)	
Smokers with mental health problems are more dependent on nicotine, % (*n*)				
Strongly disagree	2.3 (16)	2.0 (10)	3.3 (6)	χ^2^(4) = 3.2, *p* = 0.53
Disagree	5.2 (36)	4.7 (24)	6.7 (12)	
Neither agree nor disagree	12.1 (83)	12.8 (65)	10.0 (18)	
Agree	56.7 (390)	57.3 (291)	55.0 (99)	
Strongly agree	23.7 (163)	23.2 (118)	25.0 (45)	
Smokers with mental health problems are less willing to use smoking cessation medication, % (*n*)				
Strongly disagree	10.5 (72)	9.1 (46)	14.4 (26)	χ^2^(4) = 8.6, *p* = 0.072
Disagree	44.8 (308)	43.5 (221)	48.3 (87)	
Neither agree nor disagree	26.6 (183)	28.9 (147)	20.0 (36)	
Agree	16.1 (111)	16.5 (84)	15.0 (27)	
Strongly agree	2.0 (14)	2.0 (10)	2.2 (4)	
Smokers with mental health problems more often need to gradually cut down smoking before quitting, % (*n*)				
Strongly disagree	2.6 (18)	2.6 (13)	2.8 (5)	χ^2^(4) = 1.9, *p* = 0.75
Disagree	17.0 (117)	15.9 (81)	20.0 (36)	
Neither agree nor disagree	27.2 (187)	27.2 (138)	27.2 (49)	
Agree	45.6 (314)	46.9 (238)	42.2 (76)	
Strongly agree	7.6 (52)	7.5 (38)	7.8 (14)	
4-week quit outcomes are equally appropriate for smokers with mental health problems, % (*n*)				
Strongly disagree	7.8 (54)	7.1 (36)	10.0 (18)	χ^2^(4) = 3.2, *p* = 0.53
Disagree	31.1 (214)	30.1 (153)	33.9 (61)	
Neither agree nor disagree	24.6 (169)	25.4 (129)	22.2 (40)	
Agree	29.4 (202)	30.3 (154)	26.7 (48)	
Strongly agree	7.1 (49)	7.1 (36)	7.2 (13)	

Note: Bolded cells associated with adjusted residuals greater than ± 2.58 (α = 0.01).

#### 3.3.3 Confidence in supporting smokers with mental health problems

**Fig. 1:**
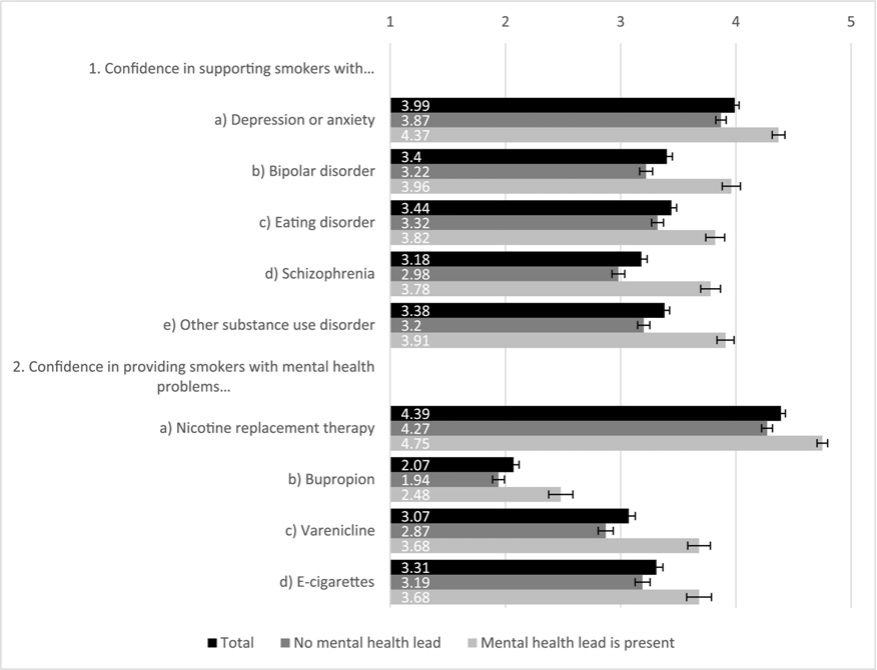
Practitioners' confidence in providing stop smoking support or medication to smokers with mental health problems (1 - not confident at all, 5 - very confident); N = 647. Note: test statistics for ‘lead’ vs. ‘no lead’ groups comparison: 1. a) *t*(391.1) = − 7.1, *p* < 0.001; b) *t*(330.9) = − 7.6, *p* < 0.001; c) *t*(301.2) = − 5.2, *p* < 0.001; d) *t*(307.3) = − 7.8, *p* < 0.001; e) *t*(333.9) = − 7.8, *p* < 0.001. 2. a) *t*(504.5) = − 7.2, *p* < 0.001; b) *t*(244.2) = − 4.7, *p* < 0.001; c) *t*(309.5) = − 6.9, *p* < 0.001; d) *t*(283.2) = − 3.9, *p* < 0.001.

Practitioners were most confident in supporting smokers with depression or anxiety disorders and least confident in aiding smokers with schizophrenia (Fig. 1). Regarding potential cessation support, practitioners were most confident recommending or providing nicotine replacement therapy (NRT) products, moderately confident with varenicline and e-cigarettes, and least confident with bupropion. Practitioners who worked in services that had a lead for mental health work were more confident than colleagues without a lead in all comparisons (all *p* < 0.001, Fig. 1).

### 3.4 Knowledge and training needs

Practitioners estimated that 41.8% (SD = 20.1, 95% CI: 40.3–43.2) of smokers in the UK and 32.2% (SD = 27.0, 95% CI: 30.0–34.4) of their clients have a current mental health problem. A third of practitioners (34.2%) disagreed with a misconception that smoking helps people with mental health problems feel better and about half (52.7%) disagreed with the misconception that smoking cessation is detrimental to mental health; this was more likely if practitioners worked in a service that had a mental health lead (χ^2^(4) = 17.9, *p* = 0.001) (Table 3). When asked about effects on blood levels of psychiatric medication, 48.3% of practitioners correctly indicated that tobacco smoking may decrease blood levels of some medication, 52.7% correctly indicated that quitting smoking may increase blood levels, and 33.4% knew that nicotine does not affect psychiatric medication metabolism. Regarding stop smoking medication cautions, almost two thirds of practitioners (63%) knew that NRT products have no cautions or contraindications related to mental health, 27.5% answered correctly that bupropion had both cautions and contraindications, and 44.6% indicated that varenicline had cautions for smokers with mental health conditions (these were removed just before data collection started).

**Fig. 2:**
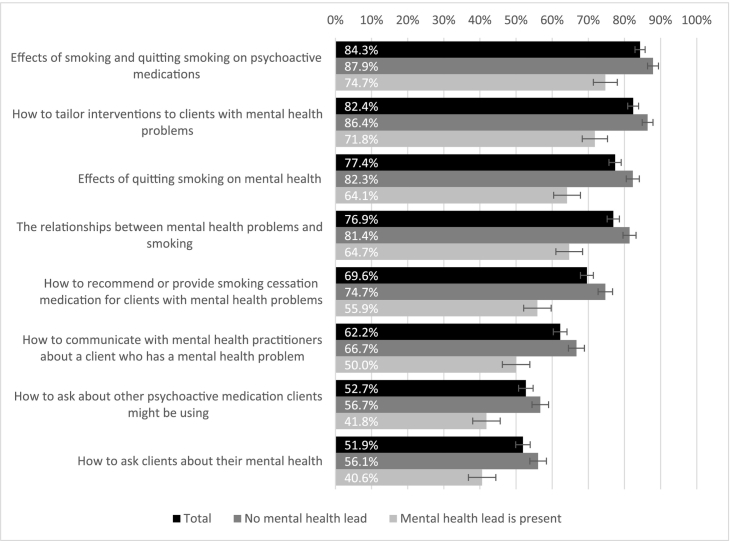
Practitioners' training needs; N = 632. Note: proportions refer to practitioners who expressed the need for specific training.

Practitioners' training needs are summarised in Fig. 2. The smallest proportion, albeit still over half of practitioners were interested in how to ask clients about mental health (51.9%) and how to ask about medication clients are prescribed (52.7%). Practitioners were most interested in what effects smoking and cessation have on psychiatric medication (84.3%) and how to tailor smoking cessation support to clients with mental health problems (82.4%). Practitioners who did not have a lead for mental health work were in general more interested in training than practitioners who had a lead (all *p* < 0.001, Fig. 2).

## 4 Discussion

Survey findings suggest that stop smoking practitioners who responded to the survey lack resources to identify and support smokers with mental health problems. Practitioners' current practice, attitudes, and knowledge on smoking and mental health are not always in line with the evidence and their confidence is moderate. Although practitioners from services with a lead for mental health work were more knowledgeable and confident in supporting smokers with mental health problems, the majority nevertheless expressed the need for training about smoking and mental health.

Our findings should be considered in light of a few limitations. Firstly, as there are no reliable ways to approach all UK practitioners who support smoking cessation, we reached our survey respondents through the database of the national smoking cessation training provider. Although we approached everyone who at some point signed up for smoking cessation training (NICE, 2008), we could not verify how many of their emails were invalid or what proportion of potential participants were currently supporting quit attempts; this also made it impossible to assess the survey response rate. However, the national regulators recommend that all practitioners in England who provide smoking cessation support are certified by the same training provider (NCSCT, 2014a, NICE, 2008), so our invitations had likely reached the vast majority of practising stop smoking practitioners. There are no national data available on the characteristics of stop smoking practitioners to determine the representativeness of our sample. Respondents were similar in demographic (gender, age) and professional (work role, work experience) characteristics to practitioners who participated in similar surveys (Brose et al., 2015, McDermott et al., 2013, McDermott et al., 2012), but the low response rate to email invitations and the threshold used to select sufficiently complete questionnaires limit the generalisability of findings to all UK stop smoking practitioners. It is likely that practitioners more interested in links between smoking and mental health were over-represented. Also, data consisted of participants' self-reported answers that were not compulsory, which resulted in different sample sizes for different questions. Finally, we could not account for how many practitioners worked in the same service or for the same employer.

In a recent survey, a majority of regional tobacco control leads in England reported undertaking targeted work to help smokers with mental health problems (ASH, 2016). Our findings suggest that services lack resources for such targeted work and this seems to persist: similar strategic barriers, including scarcity of specific funding or lack of support for mental health work from a designated staff member, were highlighted in a 2010 study of London Stop Smoking services (McNally & Ratschen, 2010). In addition to service-level barriers, support for practitioners seems insufficient, as many did not have a lead or a manual guiding work with smokers who have mental health problems. Best practice guidelines advise practitioners to routinely record clients' mental health status and medication they take, and to inform others involved in a smoker's healthcare about the cessation attempt (NCSCT, 2014a). Practitioners were least compliant with the advice to inform other healthcare specialists about a cessation attempt, indicating insufficient cooperation between stop smoking services and primary care or mental health specialists.

To the best of our knowledge, this study was the first attempt to look into how practitioners who support smoking cessation perceive and work with smokers who also have mental health problems. The data show that misconceptions about smokers with mental health problems existent among mental healthcare professionals (Sheals et al., 2016) are also found among smoking cessation practitioners. A substantial proportion of respondents were not sure or supported erroneous statements (such as stopping smoking worsens symptoms of a mental health problem), representing an important gap in knowledge, which could contribute to deficiency in routine screening of clients' mental health status and other medication they might be taking, lack of tailoring services to specific mental health conditions, and difficulties in establishing rapport with smokers who have mental health problems.

Practitioners' confidence in supporting smokers with various mental health conditions was moderate. They were least confident in helping smokers with schizophrenia, possibly due to the usually more complex support these clients require, including monitoring of psychiatric medication (Ratschen et al., 2009). This was in accord with practitioners' expressed training needs. Practitioners who were or had a service lead for mental health work were more confident in all areas than their colleagues without such support. Some encouraging findings also emerged: practitioners had confidence in their ability to support smokers with depression and anxiety, the most common mental health problems, and they were confident about using NRT with smokers who have mental health problems.

Practitioners lacked knowledge about smoking effects on blood levels of psychiatric medication and about contraindications and cautions of cessation medication. However, high interest in training about smoking effects on psychiatric medication suggests that practitioners were aware of their limited knowledge.

Services' resources to support smokers with mental health problems should be improved. Findings suggest that part of the knowledge and practice issues could be addressed by appointing a lead staff member responsible for supporting smokers with mental health problems in each service. However, active participation of a mental health lead in supporting and training staff could rectify some but not all gaps: for instance, regardless of the presence of a mental health lead in a service, similar proportions of practitioners supported the misconception that smoking helps those with mental health problems to feel better. Simultaneously, practitioners' knowledge and support for smokers with mental health problems could be improved by specifically tailored training and treatment manuals (Brose et al., 2015, Brose et al., 2014). Generally, a comprehensive theoretical assessment of existing implementation problems using an integrative theoretical framework (Cane et al., 2012, Michie et al., 2005) could be promising approach in order to improve smoking cessation support for smokers with mental health problems.

While stop smoking practitioners' skills and knowledge are crucial in tailoring support for smokers who have mental health problems, the biggest impact, however, cannot be achieved by smoking cessation services alone. These smokers are more frequently seen by primary and mental healthcare services, therefore the responsibility for addressing and treating smoking, the major cause of premature morbidity and mortality in people with mental health problems, should be with all professionals who treat mental or physical health problems.

## 5 Conclusions

Smokers who have mental health problems want and can stop smoking, and would hugely benefit from smoking cessation; however, there is a significant minority of practitioners whom this message has yet to fully reach. Practitioners supporting smoking cessation have limited knowledge of some of the main issues with smoking cessation in those with mental health problems, and are hindered by a lack of resources.

The following are the supplementary data related to this article.

Table A1 Survey questions and answers used in the study. Table A1

## Conflict of interest

AMcE has received travel funding, honorariums and consultancy payments from manufacturers of smoking cessation products (Pfizer Ltd., Novartis UK and GSK Consumer Healthcare Ltd) and hospitality from North51 who provide online and database services. He also receives payment for providing training to smoking cessation specialists and receives royalties from books on smoking cessation. AMcE is a trustee and board member of the charity Action on Smoking and Health (ASH) and an associate member of the New Nicotine Alliance (NNA), a charity that works to foster greater understanding of safer nicotine products and technologies. ES, DR and LB have no competing interests.

## Funding

This work was supported by a Cancer Research UK (CRUK)/BUPA Foundation Cancer Prevention Fellowship (C52999/A19748) awarded to LB.
