# Supplementary material for: Cessation support for smokers with mental health problems: a survey of resources and training needs
Source: J Subst Abuse Treat. 2017 Sep;80:37–44. doi: 10.1016/j.jsat.2017.06.008 (PMC5555355; doi:10.1016/j.jsat.2017.06.008)
Supplement: Table A1 — Survey questions and answers used in the study. [file mmc1.docx]

**Supplementary material for the article**

**Cessation support for smokers with mental health problems: a survey of resources and training needs**

Erikas Simonavicius^1^, Debbie Robson^1^, Andy McEwen^2^, Leonie S. Brose^1, 3^

^1^ Department of Addictions, Institute of Psychiatry, Psychology and Neuroscience, King’s College London, London, United Kingdom

^2^ National Centre for Smoking Cessation and Training, 1 Great Western Industrial Centre, Dorchester, United Kingdom

^3^ UK Centre for Tobacco and Alcohol Studies, United Kingdom

Correspondence to: Erikas Simonavicius, Department of Addictions, Institute of Psychiatry, Psychology and Neuroscience, King’s College London, 4 Windsor Walk, London SE5 8BB, United Kingdom. E-mail: erikas.simonavicius@kcl.ac.uk

**Table A1** Survey questions and answers used in the study

| **Question** | **Response options** |
| --- | --- |
| **Demographic and work characteristics** |  |
| 1. What is your gender? | Male; Female |
| 1. How old are you? | 18-25; 26-34; 35-44; 45-54; 55-64; 65 or older |
| 1. In which country do you live? | England; Scotland; Wales; Northern Ireland |
| 1. Tell us about your Stop Smoking Service work | My main role involves providing smoking cessation and behavioural support to clients wanting to quit (sometimes called a specialist practitioner/advisor); I sometimes give smoking cessation and behavioural support to clients wanting to quit but my job also involves other activities that are not to do with smoking (sometimes called a community or level 2 practitioner/advisor); I am a Stop Smoking Service manager; I am a Stop Smoking Service commissioner; Other (with an option to specify) |
| 1. Who is your employer?   (originally, the 22^nd^ item on the survey) | I am employed by the local authority; I am employed by a company that runs stop smoking services; I am employed by a charity or community interest company; I am employed by a GP practice; I am employed by a pharmacy; I am employed by a dental service; I am employed by an NHS organisation; Other (with an option to specify) |
| 1. How long have you been providing smoking cessation service as a part of your job?   (originally, the 23^rd^ item on the survey) | Drop-down menu from 0 to 20 years in 6 months increments |
| **Smoking cessation support for smokers with mental health problems** |  |
| 1. Does your Stop Smoking Service have specific team members nominated as a lead for mental health work? | Yes, that is me/I am one of them; Yes, a colleague/colleagues; No; Don’t know |
| 1. Does your Stop Smoking Service have specific funding for the provision of stop smoking support to smokers with a mental health problem? | Yes; No; Don’t know |
| 1. Does your Stop Smoking Service have a system to record clients’ mental health problem? | Yes; No; Don’t know |
| 1. Does your Stop Smoking Service have any treatment manuals or guidance documents telling you how to support clients who have a mental health problem? | Yes; No, our service has a treatment manual, but it does not address mental health; No, our service doesn’t have a treatment manual; Don’t know |
| 1. When you start working with a client, how often, if at all, do you ask about his/her mental health? | Never; Rarely; Sometimes; Very often; Always |
| 1. How often, if at all, do you record what medication (other than stop smoking) your clients are currently taking? | Never; Rarely; Sometimes; Very often; Always |
| 1. When you record information about other medication, how often, if at all, do you record the dosage your clients are taking? | Never; Rarely; Sometimes; Very often; Always |
| 1. If you are aware that a client has a mental health problem, how often, if at all, do you contact his/her GP/mental health services about the smoking cessation attempt? | Never; Rarely; Sometimes; Very often; Always |
| **Practitioners’ attitudes and confidence in supporting smokers with mental health problems** |  |
| 1. Compared with smokers without mental health problems… 2. Smokers with mental health problems are less interested in stopping smoking 3. Smokers with mental health problems are less successful in quitting smoking 4. Smokers with mental health problems are more dependent on nicotine 5. Smokers with mental health problems are less willing to use smoking cessation medication 6. Smokers with mental health problems more often need to gradually cut down smoking before quitting 7. 4-week quit outcomes are equally appropriate for smokers with mental health problems | Strongly disagree; Disagree; Neither agree nor disagree; Agree; Strongly agree |
| 1. Please indicate on a scale from 1 to 5 (1 – not at all confident, 5 – very confident) how confident you feel in:   Providing smoking cessation support for clients with…   1. Depression or anxiety 2. Bipolar disorder 3. Eating disorder 4. Schizophrenia 5. Other substance use disorder   Recommending or providing the following medication for clients with any mental health problem…   1. NRT 2. Bupropion (Zyban) 3. Varenicline (Champix) 4. Electronic cigarettes | Scale from 1 (not at all confident) to 5 (very confident) |
| **Difficulties supporting smokers with mental health problems** |  |
| 1. Have you ever experienced any difficulties recommending or providing stop smoking medication for clients with mental health problems? If yes, please briefly describe them | Free text response |
| **Knowledge and training needs** |  |
| 1. To what extent do you agree or disagree with the following statements? |  |
| 1. Smoking helps those with a mental health problem to feel better | Strongly disagree; Disagree; Neither agree nor disagree; Agree; Strongly agree |
| 1. Stopping smoking worsens symptoms of a mental health problem | Strongly disagree; Disagree; Neither agree nor disagree; Agree; Strongly agree |
| 1. What percentage of all smokers in Great Britain would you say have a current mental health problem? | Drop-down menu from 0% to 100% in 5% increments |
| 1. What percentage of your clients (how many out of 100 that you see) are referred from mental health treatment services (community mental health teams, psychiatric wards, etc.)? | Drop-down menu from 0% to 100% in 5% increments |
| 1. Please indicate whether the following behaviours are likely to decrease, not affect, or increase blood levels of some psychoactive medication (clozapine, tricyclic antidepressants, etc.) 2. Tobacco smoking 3. Quitting smoking 4. Using nicotine (nicotine patch, electronic cigarette, etc.) | Decreases the blood level of psychoactive medication; Does not affect the blood level of psychoactive medication; Increases the blood level of psychoactive medication; Don’t know |
| 1. Please indicate whether you think that these smoking cessation medication have any contraindications (are not suitable) and/or warnings (have to be used with caution) when prescribed to clients with a mental health problem 2. NRT 3. Bupropion (Zyban) 4. Varenicline (Champix) | Contraindication(s); Warning(s); Both; Neither; Don’t know |
| 1. I would like more training on 2. How to ask clients about their mental health 3. How to ask about other psychoactive medication clients might be using 4. How to communicate with mental health practitioners if a client has a mental health problem 5. The relationships between mental health problems and smoking 6. Effects of smoking and quitting smoking on psychoactive medications 7. Effects of quitting smoking on mental health 8. How to recommend or provide smoking cessation medication for clients with a mental health problem 9. How to tailor Stop Smoking interventions for clients with a mental health problem | Yes; No |
